# Tracking emergency response actions during COVID-19 leads to development of an innovative public health evaluation tool

**DOI:** 10.17269/s41997-023-00811-3

**Published:** 2023-08-07

**Authors:** Suzanne Biro, Karen Scott, Emma Nagy, Nancy Slipp, Kinsey Beck, Christina Catley, Ezra Hart

**Affiliations:** 1 KFL&A Public Health, Kingston, ON Canada; 2 Canadian Institute for Health Information, Ottawa, ON Canada

**Keywords:** Public health, COVID-19, Non-pharmaceutical interventions, Evaluation, Qualitative research, Data visualization, Santé publique, COVID-19, interventions non pharmaceutiques, évaluation, recherche qualitative, visualisation de données

## Abstract

**Setting:**

Early in the pandemic, KFL&A Public Health needed a way to capture, organize, and display COVID-19-related events to be accountable for and evaluate our actions.

**Intervention:**

We used accessible software (Microsoft Office 365 suite, Microsoft PowerBI) to develop a data collection and visualization system. The Canadian Institute for Health Information (CIHI) developed a timeline and categorization approach for provincial and national COVID-related interventions, which was used to develop a regional version for local events using similar categories. We collected and displayed qualitative data alongside epidemiological data that allowed users to display different timelines of actions and outcomes and evaluate our response.

**Outcomes:**

In developing the timeline, we took stock of the information and data we wanted to collect, sort, and display locally. Next, we collected information on response actions, case and contact tracing, and staffing changes in a database that we displayed on a timeline. We included CIHI’s data set to provide insight into pandemic response across all jurisdictions.

**Implications:**

Our timeline tool has many advantages for public health authorities beyond responding to a rapidly evolving emergency. By collecting information on events as they occur, decisions and actions are documented that may otherwise be overlooked. This enables decision-makers to visualize the impact of public health actions on health outcomes over time. The tool is completely customizable and scalable depending on the project scope and we plan to apply this method to other public health programming. Finally, we include lessons learned from quickly developing these tools in a real-time pandemic setting, both locally at KFL&A Public Health and nationally at CIHI.

## Résumé

**Lieu:**

Au début de la pandémie, le Bureau de santé de Kingston, Frontenac, Lennox et Addington (KFL&A) avait besoin d’un moyen de saisir, d’organiser et de présenter les événements liés à la COVID-19 pour pouvoir en rendre compte et évaluer ses actions.

**Intervention:**

Nous avons utilisé des logiciels accessibles (Microsoft Office 365, Microsoft PowerBI) pour mettre au point un système de collecte et de visualisation de données. L’Institut canadien d’information sur la santé (ICIS) a créé un fil d’actualité et une approche de catégorisation pour les interventions provinciales et nationales liées à la COVID; nous avons créé une version régionale de ces outils pour présenter les événements locaux, en utilisant des catégories semblables. Nous avons collecté et affiché des données qualitatives en plus des données épidémiologiques, ce qui a permis aux utilisateurs d’afficher les mesures prises et leurs résultats sur différentes périodes et d’évaluer leurs interventions.

**Résultats:**

Pour créer ce fil d’actualité, nous avons fait l’inventaire des informations et des données que nous voulions collecter, trier et présenter localement. Ensuite, nous avons réuni des informations sur les mesures d’intervention, les enquêtes sur les cas, la recherche de contacts et les changements de personnel dans une base de données, et nous les avons présentées dans un fil d’actualité. Nous avons inclus le jeu de données de l’ICIS pour apporter un éclairage sur la lutte contre la pandémie dans toutes les administrations du pays.

**Conséquences:**

Notre fil d’actualité présente de nombreux avantages pour les autorités de santé publique, en plus de la possibilité de réagir à une situation urgence qui évolue rapidement. En collectant des informations sur les événements à mesure qu’ils se produisent, il est possible de documenter des décisions et des mesures qui risquent d’être oubliées sinon. Cela permet aux décideurs de visualiser dans le temps l’effet des mesures de santé publique sur les résultats cliniques. L’outil est entièrement personnalisable et évolutif, selon la portée du projet, et nous avons l’intention de l’appliquer à d’autres programmes de santé publique. Enfin, nous présentons les leçons de la mise au point rapide d’un tel outil pendant une pandémie en temps réel, tant à l’échelle locale (à la Santé publique de KFL&A) qu’à l’échelle nationale (à l’ICIS).

## Introduction

Since the first Canadian reported case of coronavirus disease 2019 (COVID-19) in January 2020, the pandemic sparked extensive public health interventions to curb impacts to Canadians, the health care system, and the economy. Though all Canadian provinces and territories implemented emergency response actions, including non-pharmaceutical interventions (NPIs) (any type of health intervention not primarily based on medication, e.g., public health orders or recommendations related to physical distancing, testing, contact tracing, personal protective equipment, quarantine) and other actions taken by an organization or government body (e.g., staff redeployment, medical directives, vaccine distribution, risk communication), there was wide variation in terms of duration, approach, and geographic application, both before and after vaccination was introduced (Hale et al., 2021). Recognizing that epidemiological data, viral transmission, population characteristics, and public compliance factors vary, and that the authority and responsibility for implementing public health response actions fall to national, provincial, territorial, and local levels, the diverse application of NPIs was anticipated (Henry, 2019). Missing was an ability to track and monitor dynamic jurisdictional, multiple, and specific measures of NPIs in relation to a diverse and rapidly changing epidemiological context.

To address the urgent need for local operational intelligence and accountability, we describe the unique approach taken through a collaboration between the Canadian Institute for Health Information (CIHI) and a local Ontario public health agency, the Kingston, Frontenac and Lennox & Addington Public Health Unit (KFL&A PH), to design and implement a locally responsive data gathering, analysis, and visualization tool using both qualitative and quantitative data sources. Many NPI data collection initiatives emerged in recent years as governments around the world strove to refine emergency responses by using data to model the efficacy of different intervention strategies (Bozorgmehr et al., 2021; McCoy et al., 2020; Suryanarayanan et al., 2021). Capturing local-level NPIs has been less common, despite jurisdictional authority at this level. Importantly, using systematic categorization and data capture to measure the nuanced variation of contextual qualitative information (i.e., key communication messages, collaborations between local partners, infection prevention and control training, and resource support for community stakeholders across sectors such as schools, long-term care homes, correctional institutions, homeless shelters) is needed to answer important practice-based research and evaluation questions to inform and guide local-level capacity and resources at the front line. We approached the topic of describing a data repository tool that integrates local-level contextual and surveillance information from a practical standpoint to highlight how the tool was created, and the challenges and lessons learned, and to promote the use of a similar approach for tracking and monitoring the response to other complex public health issues.

## Setting

KFL&A PH is one of thirty-four local public health units (LPHUs) in Ontario, Canada. LPHUs have direct responsibility for the administration of public health programs and services, including a large part in the prevention and control of the spread of COVID-19 in the community (Association of Local Public Health Agencies, 2022; Plante et al., 2023). KFL&A PH serves an urban-rural population of 209,230 (2020) located in southeastern Ontario. The City of Kingston accounts for 64.8% of the population at 135,707. A collaboration with CIHI was integral to the development of the data repository tool and the KFL&A Pandemic Timeline. CIHI is an independent, not-for-profit organization that delivers comparable and actionable information to accelerate improvements in health care, health system performance, and population health across the continuum of care. Between January 2020 and October 2021, KFL&A PH adapted CIHI’s data infrastructure and methodology to apply to our own jurisdiction.

## Issue

Implementation of NPI policies across Canada varied according to jurisdictional authority (i.e., federal, provincial and territorial, sub-provincial or regional, and municipal or local), timing, duration, geography, and risk of infection (Hale et al., 2021, McCoy et al., 2020). Amid this complex and dynamic context, KFL&A PH needed a way to capture, organize, track, and display the local-level infection risk, and the contextual, historical, and resource capacity to be accountable for, and evaluate, local-level emergency response actions.

## Response

Over the course of the development of the Canadian COVID-19 Intervention Timeline, CIHI created a categorization approach for provincial and national COVID-related interventions (Canadian Institute for Health Information, 2022). KFL&A PH used this national intervention timeline as a model for inspiration to develop a regional version for local events using similar categories.

**Fig. 1:**
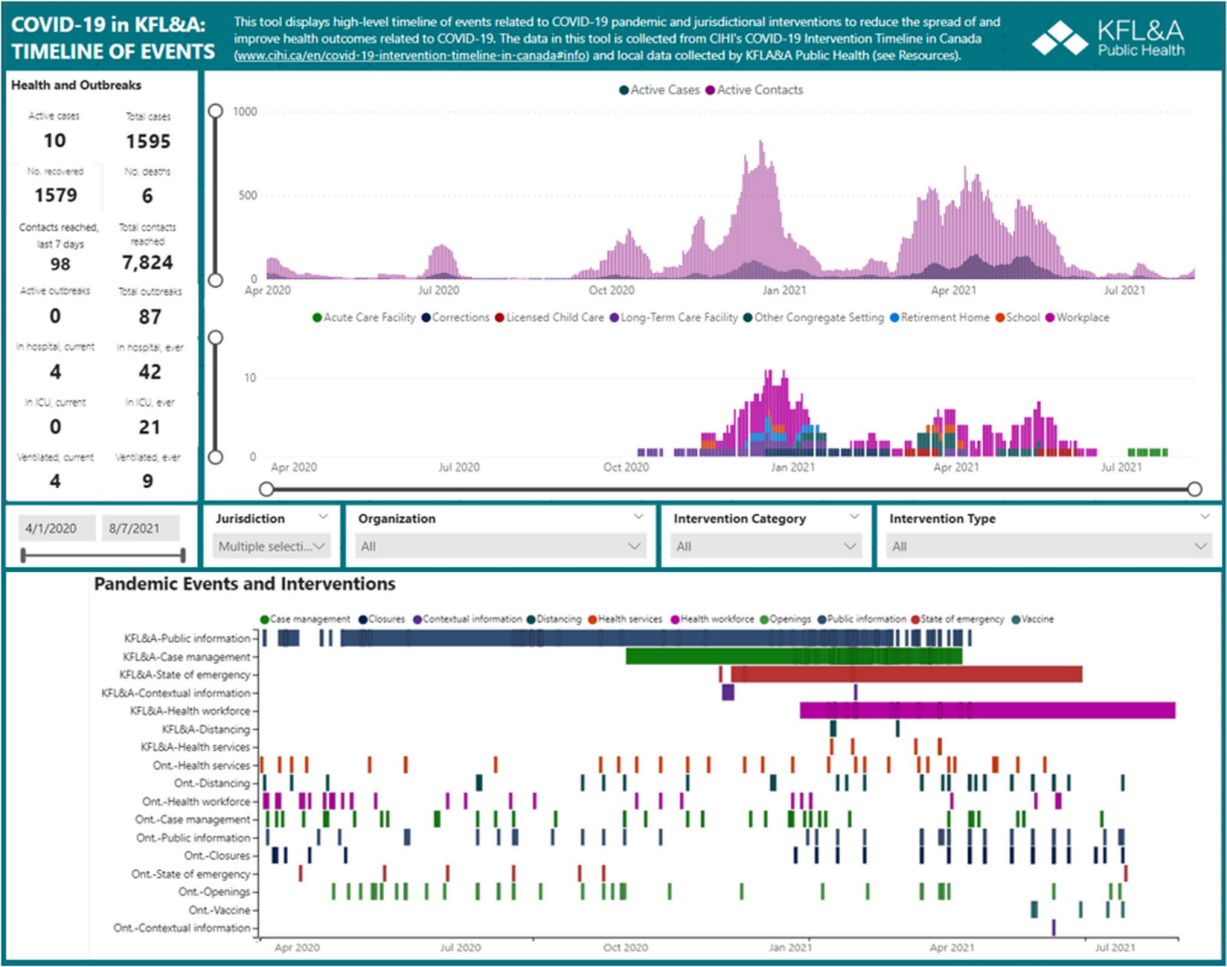
KFL&A Pandemic Timeline, displaying COVID-19 cases and contacts and locations of outbreaks along with emergency response actions, April 2020 to August 2021

Like so many organizations, KFL&A PH quickly adapted to an early COVID-19-related NPI (i.e., work-from-home orders and policies) and adopted virtual capabilities (i.e., Microsoft 365 tools). Readily available Microsoft (MS) software was leveraged to develop a data collection and visualization system, known as the KFL&A Database of Local Pandemic Response History and the Pandemic Timeline. It served as a digital repository from which contextual information and surveillance data were pulled to create a visualization tool of local-level NPIs and emergency response actions layered with epidemiological curves, as shown in Fig. 1. It is important to note the methodology continually evolved amid the rapidly fluctuating nature of the pandemic.

The primary objectives of the KFL&A Pandemic Database and Timeline were to:

- Document ongoing public health situational assessment and emergency response actions to the COVID-19 pandemic in the KFL&A region for the duration of the pandemic,
- Provide an ability to visualize information in close to real time to inform operational and resource capacity decisions,
- Be a data repository to enable local-level evaluation and inform any after-action reviews,
- Be a document repository to support any legal or medical accountability and compliance, as well as satisfy any audit requests, and
- Document the historic actions taken during the most consequential pandemic in the last 100 years from a local public health perspective.

A project team comprised of research associates, a data analyst, an epidemiologist, a public health librarian, a project manager, and a foundational standard specialist at KFL&A PH conducted the data collection and document review and collation to create the visualization tool.

A channel in MS Teams served as the digital repository to house knowledge products created with relevant MS applications related to the project as follows:

1. **Database of Local and Provincial Pandemic Response History:** a SharePoint List of emergency response actions at the regional and provincial levels served as a standardized data extraction form to record contextual information systematically for each response action.
2. **KFL&A PH Pandemic Timeline:** a timeline visualization of local and provincial emergency response actions depicted alongside surveillance data. The PowerBI-based visualization was the primary knowledge product of the project.
3. **KFL&A PH Record of Pandemic Notebook:** a digital report providing a complete overview of the project, structured off the WHO COVID-19 Strategic Preparedness and Response – Monitoring and Evaluation Framework (World Health Organization 2020). MS OneNote housed the project methodology and data dictionary.
4. **Weekly COVID-19 Unit Update**: an MS Forms questionnaire completed by COVID-19 Unit supervisors to capture each unit’s emergency response actions.
5. **Files**: documents (e.g., medical officer of health directives, long-term care home training resources) from which relevant emergency response actions were gleaned.
6. **Wiki:** includes project notes for readers and users of the project knowledge products, a record of major methodological decisions, project team meeting minutes, and a list of relevant links to important timelines and resources.

**Fig. 2:**
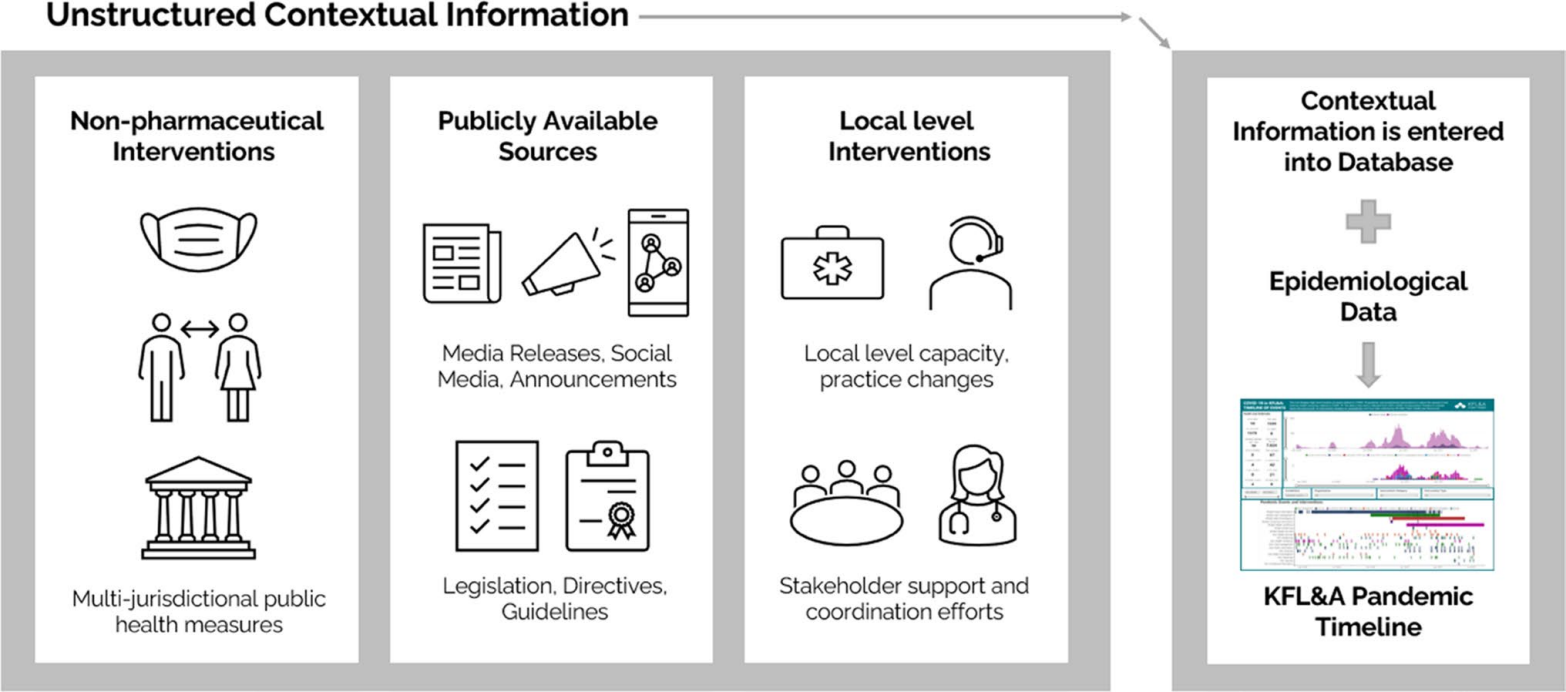
Data sources and flow to create KFL&A Pandemic Timeline (adapted from Suryanarayanan et al., 2021)

The project team used the MS Teams channel for ongoing communications, creating task lists, and tracking the progress of data collection, review, and analysis **(**Fig. 2**)**.

## Data sources

We collected information about emergency response actions and contextual qualitative and epidemiologic data from primary and secondary data sources. We reviewed and extracted information from pertinent data sources (identified below) to catalog interventions that aimed to reduce the spread and improve health outcomes related to COVID-19.

Primary sources of data included:

- Informal observational notes recorded during daily clinical morning meetings
- Completed Weekly COVID-19 Unit Update questionnaires
- Organizational documents (e.g., decision trees, process maps)
- Emails forwarded to project team from directors, managers, leads, and staff (e.g., emails related to staff capacity, practice changes)
- KFL&A Public Health announcements or notices, media releases, social media strategies, ‘Ask the MOH’ videos, and enforcement actions
- Announcements and information from provincial Ministry websites, including the Office of the Chief Medical Officer of Health (e.g., press releases, medical directives, framework documents).

Secondary sources of data included:

- References or links from primary source documents (e.g., releases from official websites, provincial or local news agencies) that provide more information not covered in the primary source collection
- Information from the Public Health Ontario website, including guidance documents, research review summaries, and other select documents
- Provincial regulatory bodies for health professionals (websites, announcements).

Epidemiologic data, including the number of active and new cases, number of active and new contacts, and number of cases hospitalized, in the ICU, or on ventilators, were collected using the Case and Contact Management System (CCM) administered by the Province of Ontario via the Ontario Laboratory Information System (Ministry of Health, 2022). The number of active outbreaks was compiled both from CCM and from COVID-19 case management databases administered by KFL&A PH for regional assessment and management of outbreaks at various congregate settings (e.g., long-term care homes).

## Data collection

The project team was divided into two groups, one to collect, review, and analyze contextual qualitative data, and a second to collect, review, and analyze epidemiologic data. We defined emergency response actions as any COVID-19-related publicly announced program, statement, media release, enforceable order, initiative, NPI, or operational change originating from KFL&A Public Health and any COVID-19-related publicly announced statement, guidance document, or medical directive from the Government of Ontario. A data dictionary, modeled from CIHI, was created to characterize and code these response actions to capture contextual information in response to COVID-19. Table 1 provides the essence of the data dictionary. The full data dictionary can be provided on request.

**Table 1:** Characteristics of the contextual emergency response actions recorded in the Database of Local and Provincial Pandemic Response History

	Variables in database	Definition
Variables that describe the response	Response	Brief description of the response, intervention, action, or announcement
	Jurisdiction	Jurisdiction covered by the response (e.g., City of Kingston, KFL&A region, provincial)
	Response category	A classification of possible responses (e.g., closures/openings, health services, public information, case management)
	Response type	A sub-classification within each response category (e.g., within closures/openings: daycares, education, non-essential services; within public information: external risk communication, internal communication, partner engagement)
	Organization	Name of the organization that made the response
	IMS* function or COVID-19 unit *Incident management system	KFL&A PH specific. Which function or unit was managing or involved with the response?
	Start date	Start/effective date of response (could be NA)
	End date	Completion/stop date of response (could be NA)
	Reopening phase	Provincial-level classification of lockdown and reopening framework
	Notes	Additional notes to further contextualize the response description
Variables to assist the project team with file location and follow-up	Documentation location	In files (unlinked), Linked**, Chase Up, NA. (‘Chase Up’: when project team knew there was documentation but had to locate it)
	Link**	URL or file pathway for pertinent document
	Status of the response	Completed, pending, unresolved. (In internal documentation (e.g., meeting minutes), it was sometimes unclear whether the response had happened or was going to happen. This variable helped the project team to track this.)

Where applicable, emergency response action categories and types were assigned a label that aligned with the CIHI intervention categories used in their environmental scan. See Table 1 under response category and response type for examples.

Given the shifting nature of the pandemic response, the list of categorical classifiers was iteratively expanded and adjusted as novel classes of response actions were identified, resulting in a total of 10 categories and 25 types.

Emergency response actions collected for this project included major local-level actions, such as:

- Any decisions related to staff capacity implications, e.g., staff scheduling change and increase/decrease staff
- Any practice changes, e.g., key messages change to align with new guidance, updated process, standard operating procedures, and flow diagrams
- Any consultations provided, actions taken, and/or coordination activities to support community stakeholders in prevention or management of COVID-19
- Any communications to community stakeholders and/or the public, e.g., letter, local memo, media release, and legal enactment announcements.

Response actions were recorded only for the administrative level for which they were implemented (e.g., provincial interventions were entered under provincial jurisdiction and would be inclusive of KFL&A PH jurisdiction). If a response action was modified, an “end date” was added for the original version recorded as a novel response action. National- and international-level pandemic response interventions or actions were considered out of scope for this project.

To ensure data extraction consistency and to add to and adjust the categorical classifiers, a subgroup of the project team met regularly to discuss data collection and review definitions of the categories. The aim was to monitor the scope of the response actions being collected and maintain accurate categorization definitions.

## Outcomes

The KFL&A Pandemic Timeline was populated with retrospective data beginning August 2020 (January 2020 to August 2020) and near-real-time data from August 2020 to January 2021. With Health Canada’s approval and distribution of vaccinations early in 2021, data collection and entry were severely compromised as KFL&A and other health units pivoted to planning and implementation of immunization strategies (also entered as contextual qualitative data in the Timeline tool). Though competing priorities hindered capacity to maintain the KFL&A Pandemic Timeline, data entry continued as able until October 2021 (at this point, staff were redeployed to manage vaccine distribution).

The database and timeline were used to extract and collate documents, confirm the timing and duration of public health measures, and provide information to satisfy requests from the Office of the Auditor General of Ontario related to COVID-19 and long-term care (Office of the Auditor General of Ontario, 2021). Additionally, the Timeline tool regularly informed Board of Health reports, two annual reports (2020 and 2021), and served to guide ongoing public health messaging and campaigns.

## Implications

There is growing recognition of the need to incorporate complex and intersecting contextual information known to influence health outcomes alongside monitoring and surveillance data. Summarizing and visualizing diverse information reveal patterns and connections more easily. Unfortunately, the full applicability of the Timeline tool and the rich repository of data have yet to be fully realized, but the patterns and connections would enable description of the relationship between the interventions and changes in the pandemic.

At the time of writing, Ontario LPHUs were only just emerging from pandemic response into a recovery phase. It is hoped the KFL&A Pandemic Timeline tool will be used to inform after-action reviews and myriad evaluation questions that will enable increased resiliency and preparation for future emergency response.

## Lessons learned

Due to the innovative nature and requirement of fast-paced development, both KFL&A PH and CIHI were learning and adapting their projects as they went. Though there were three knowledge exchange meetings, each organization implemented their projects independently, KFL&A PH tailoring their scope to the local level and CIHI reporting at provincial, territorial, and national levels. Despite the different scales of the projects (national versus local), challenges were similar:

- The amount and pace of new information being produced amid the evolving nature of the pandemic, and public health’s responses, stretched abilities to keep pace with data collection. Future projects of this nature would benefit from built-in processes for data collection with clear protocols and the possible use of artificial intelligence (AI). Also, it is recommended that both methods and scope be reviewed regularly.
- The variation in terminology used between jurisdictions and different information sources required creative search strategies to ensure all pertinent information was collected. Though difficult to get buy-in, having a centralized location to disseminate information and encouraging standardized language and phrasing of key concepts across jurisdictions would simplify data analysis and reduce the amount of editing required.
- Qualitative data are rich with context and nuance. However, these qualities pose a challenge to the consistency and accuracy of data entry. Though both KFL&A PH and CIHI limited the number of people entering data, the need for quality checks to ensure consistency of language and accuracy of information remained. Data validation exercises and consistency checks should be incorporated into data entry processes to ensure reliable entries.
- KFL&A PH and CIHI had different experiences with the software they used for their timeline visualization. KFL&A PH used a product they had access to in-house (i.e., PowerBI), whereas CIHI worked with a vendor to create their visualization. Each had its drawbacks. While an in-house product meant there was flexibility to modify the display in a timely fashion, the software is less sophisticated; the functionality of the timeline was not as extensive as the CIHI timeline. Selecting software, of course, will be based on more than flexibility and functionality; cost, sharing capability, and expertise required for its use should also be considered.

## Implications for policy and practice

What are the innovations in this policy or program?

- Systematic categorization to capture the variety of contextual qualitative information at different jurisdictional and operational levels.
- A comprehensive picture of the status of emergency response actions in relation to epidemiological curves, informed by contextual qualitative and quantitative data sources.
- Creation of a locally responsive data gathering, analysis, and visualization tool that could be populated by different users to ease the burden of real-time data entry.
- Data collected can be used to track the impact of emergency response actions (and potentially other complex public health interventions, e.g., policy development) in a community over time.
- Data collected can be analyzed and used in different ways, by different people or partners to inform near-real-time decision-making; support collaborative community emergency response strategies using a virtual, shared platform; appreciate and account for multiple and intersecting factors influencing situational assessment; cross-validate NPI patterns, temporal sequences, and possibly spatial patterns; contribute local-level data to multiple research questions related to the impacts of specific measures on the pandemic across different geographical regions (i.e., local and provincial) or population sub-groups, as well as associations between levels; and inform after-action reviews and answering multiple evaluation questions.

What are the burning research questions for this innovation?

- Using the tool, how do the recorded response actions seem to influence the undulations of the pandemic activity from 2020 to 2022?
- How can the categorization methodology be used in other applications where it is difficult to categorize public health actions so that they can be aligned with other actions and actors also responding to a crisis?
- How can this methodology be used to incorporate the rich and complex contextualizing factors and intersectional experiences related to the social determinants of health, the structural drivers of health (e.g., racism, colonialism, capitalism, Indigenous worldviews) alongside surveillance data?
- Can this type of tool provide systems-level evidence to match the complexity of many challenging public health commitments: policy development, promotion of mental health and well-being, chronic disease prevention, climate change mitigation?
- Does this tool enable collaborative data entry capacity—once scaffold definitions and protocol are established—to ease the burden while continuing to depict a picture that illuminates patterns and connections from a bird’s eye view?

## Data Availability

Not applicable
